# Nonstationary footprints of ENSO in the Mekong River Delta hydrology

**DOI:** 10.1038/s41598-022-20597-7

**Published:** 2022-12-07

**Authors:** Takaaki K. Watanabe, Tung Thanh Phan, Atsuko Yamazaki, Hong-Wei Chiang, Chuan-Chou Shen, Lam Dinh Doan, Tsuyoshi Watanabe

**Affiliations:** 1 Department of Natural History Sciences, Faculty of Science, Hokkaido University, Sapporo, 060-0810 Japan; 2 KIKAI Institute for Coral Reef Sciences, Kikai Town, Kagoshima, 891-6151 Japan; 3 Institut für Geowissenschaften, Christian-Albrechts Universität zu Kiel, 24118 Kiel, Germany; 4 Faculty of Geology, University of Science, Vietnam National University, Hanoi, 334 Nguyen Trai Street, Thanh Xuan District, Hanoi, Vietnam; 5 Department of Earth and Planetary Sciences, Faculty of Science, Kyusyu University, Fukuoka, 819-0395 Japan; 6 Research Center for Environmental Change, Academia Sinica, Taipei, 11529 Taiwan, ROC; 7 High-Precision Mass Spectrometry and Environment Change Laboratory (HISPEC), Department of Geosciences, National Taiwan University, Taipei, 10617 Taiwan, ROC; 8 Research Center for Future Earth, National Taiwan University, Taipei, 10617 Taiwan, ROC; 9 Department of Sedimentary Geology, Institute of Geological Sciences, Vietnam Academy of Science and Technology, 18 Hoang Quoc Viet, Hanoi, Vietnam

**Keywords:** Climate sciences, Hydrology, Limnology, Palaeoceanography, Palaeoclimate

## Abstract

The Mekong River Delta (MRD) is an essential agricultural area for the worldwide rice supply. Floods and droughts triggered by El Niño southern oscillation (ENSO) have been threatening sustenance in the MRD. Sustainable food supplies require understanding the response of the MRD hydrology to the changing ENSO behaviour in recent decades. Here, we reconstructed the annual rainfall maxima in the MRD using the oceanic paleoclimate proxy from coral skeletons and compared them with ENSO indexes. Annual minima of coral-based seawater oxygen isotope (δ^18^O_sw_) correlated with annual rainfall maxima, which allowed to extend rainfall data from 1924 to the recent. The annual rainfall maxima based on δ^18^O_sw_ negatively correlated with the central Pacific El Niño index. This suggested that La Niña and central Pacific El Niño events lead to heavy and light rainy seasons. The heavy rainy season had more serious impacts in recent decades, which likely increases the flood risk. In contrast, the frequency and rainfall amount of the light rainy season has not changed significantly, although a catastrophic drought has hit the MRD. Our finding concludes that the impact of the ENSO event on MRD hydrology is inconsistent in the past century.

## Introduction

Agriculture in the Mekong River Delta (MRD) is the key to sustaining the worldwide food supply. Vietnamese rice production contributed 7.5% of the total global rice exports in 2017 CE, which was supported by its production in the MRD^1,2^. However, recent severe hydrological disasters with frequent floods and droughts threaten the MRD^2,3^. Flood events seriously impact agriculture and health hazards since the large cities and agricultural fields in the MRD are at low elevations^4^. Drought events, such as those in 2015 and 2019 CE, cause collapse agricultural systems by causing moisture loss from the soil and intruding saline groundwater into agricultural fields^5^. Over 4600 km^2^ of rice field was damaged, and 200,000 households suffered from safe water shortages in 2019 CE^6^. Understanding the dynamics of rainfall changes and the Mekong River flow rate is crucial for sustainable food supply^7–9^.

Previous studies^10,11^ approached the hydrological changes in the MRD using the observation and land-based proxy records of rainfall in the Indochina peninsula (e.g., tide gage, tree rings, observatory data^10–15^). The El Niño Southern Oscillation (ENSO) has an important role in river runoff and rice production in the MRD, as El Niño (La Niña) events would induce the short (long) wet season with a low (high) amount of rainfall^2,15–17^. The light rainy season during El Niño allows intrusions of saline water^16^. The heavy rainy season causes flood events^16^. Food sustainability requires how the hydrology event in MRD responds to the ENSO in the future warming earth^2^, as the ENSO behaviour has been changing in recent decades^18^. However, rainfall observations are spatiotemporally heterogeneous. Most of the proxy records and tidal data are far from the river mouth of the MRD^13,19,20^. These limitations of available records and the spatial heterogeneity of rainfall make it complex to reveal the past hydrological changes of the MRD. Here we investigate the frequency and intensity of hydrological changes during the last century using a coral proxy record. The chemical composition in a drilled coral core allows us to calculate an oxygen isotopic ratio in seawater (δ^18^O_sw_, a proxy for hydrology changes^21–23^) which enables a reliable reconstruction of paleolimnology^24^ and palaeoceanography^25,26^. Reconstruction of the interannual to decadal hydrological change using the oceanic proxy could imply mechanisms behind the flood and drought events with the anthropogenic^27^ and natural^12,13^ hydrological changes.

**Figure 1:**
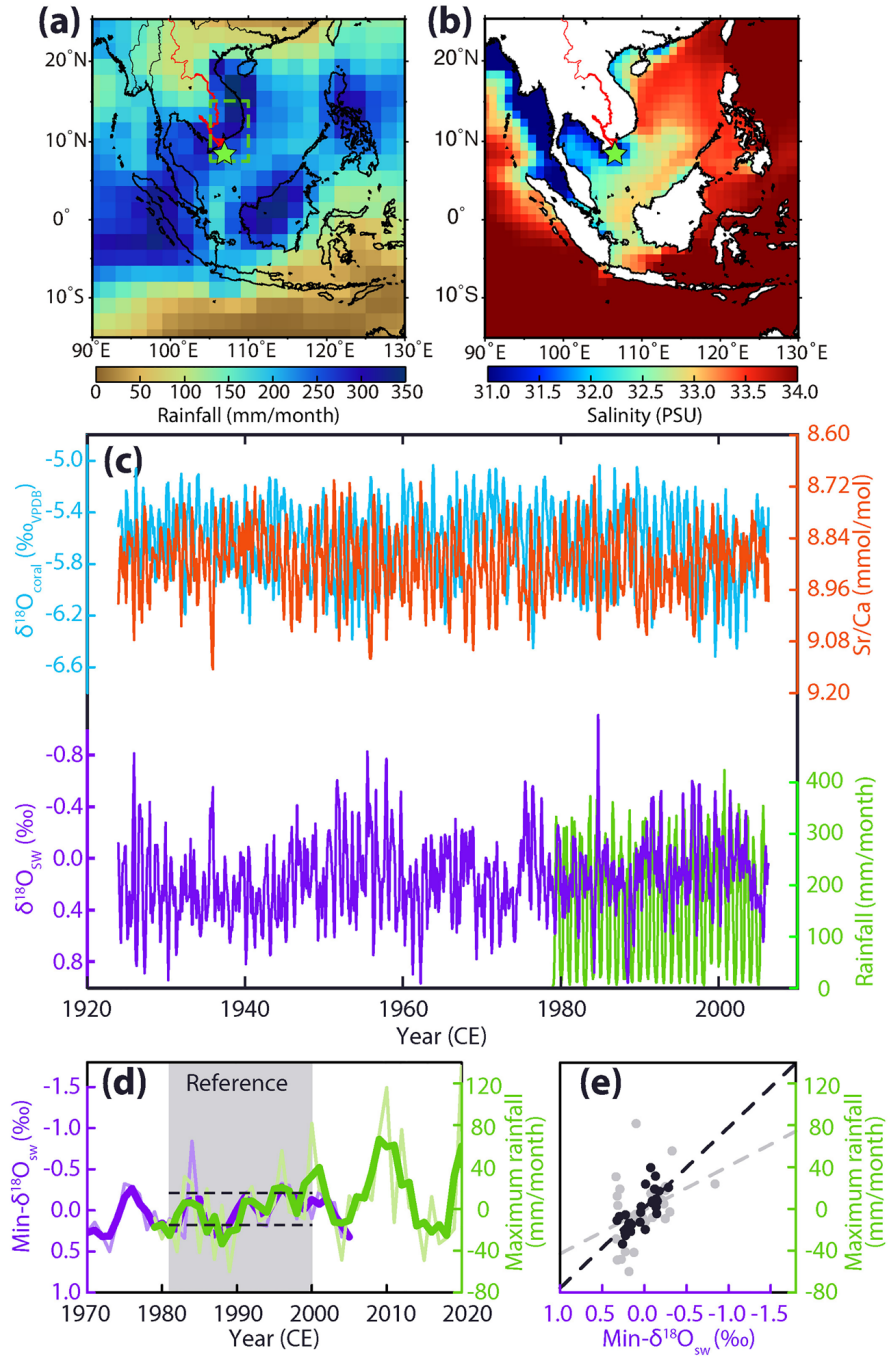
Calibration of coral-based records with meteorological data. (**a**,**b**) Rainfall (GPCP version 2.3) and salinity (EN4.2.1^45^) in October from 1980 to 2005 CE. The star symbol indicates Con Dao Island. The red line shows the Mekong River. The map was generated by the GMT 4.5.12^46^. (**c**) Monthly resolution coral records of δ^18^O_coral_ (blue), Sr/Ca ratio (red), and δ^18^O_sw_ (purple) from December 1923 to June 2006 on Con Dao Island. GPCP version 2.3 rainfall from 1979 to 2020 (green) was derived from the southeastern part of the Indo-China Peninsula (7.5–15°N, 105–110°E, green box in panel **a**). (**d**) Min-δ^18^O_sw_ (purple) and maximum rainfall_GPCP_ in the MRD (green). Thin and thick lines show the annual and 3-year moving averaged data, respectively. Grey shading and dashed lines indicate the reference interval (1981–2000 CE) and thresholds used to define heavy/light rainy seasons. (**e**) A cross plot analysis between the min-δ^18^O_sw_ and the maximum rainfall_GPCP_ (grey: annual data; black: 3-year moving averaged data). Dashed lines indicate linear regression lines.

We drilled a coral core (CD-4, *Porites.* sp, 150 cm in length, Fig. S1) on 8^th^ June 2006, from the southwestern coast of Con Dao Island (8° 33′ 25.7″ N, 106° 33′ 00.7″ E, ca. 90 km south of the MRD, Figs. 1a, b, and S2)^28^. Monthly resolved subsamples were obtained from sliced coral slabs for measurements of Sr/Ca (a proxy for sea surface temperature, SST^29^) and oxygen isotope ratio in coral skeletons (δ^18^O_coral_) (see Methods). The δ^18^O_sw_ was calculated from the measured δ^18^O_coral_ by subtracting the temperature component inferred from Sr/Ca using the centring method^30^. We took the annual minima of δ^18^O_sw_ (min-δ^18^O_sw_) as an indicator for maximum freshening. The min-δ^18^O_sw_ was reported as an anomaly relative to the mean during the reference period (1981–2000 CE).

**Figure 2:**
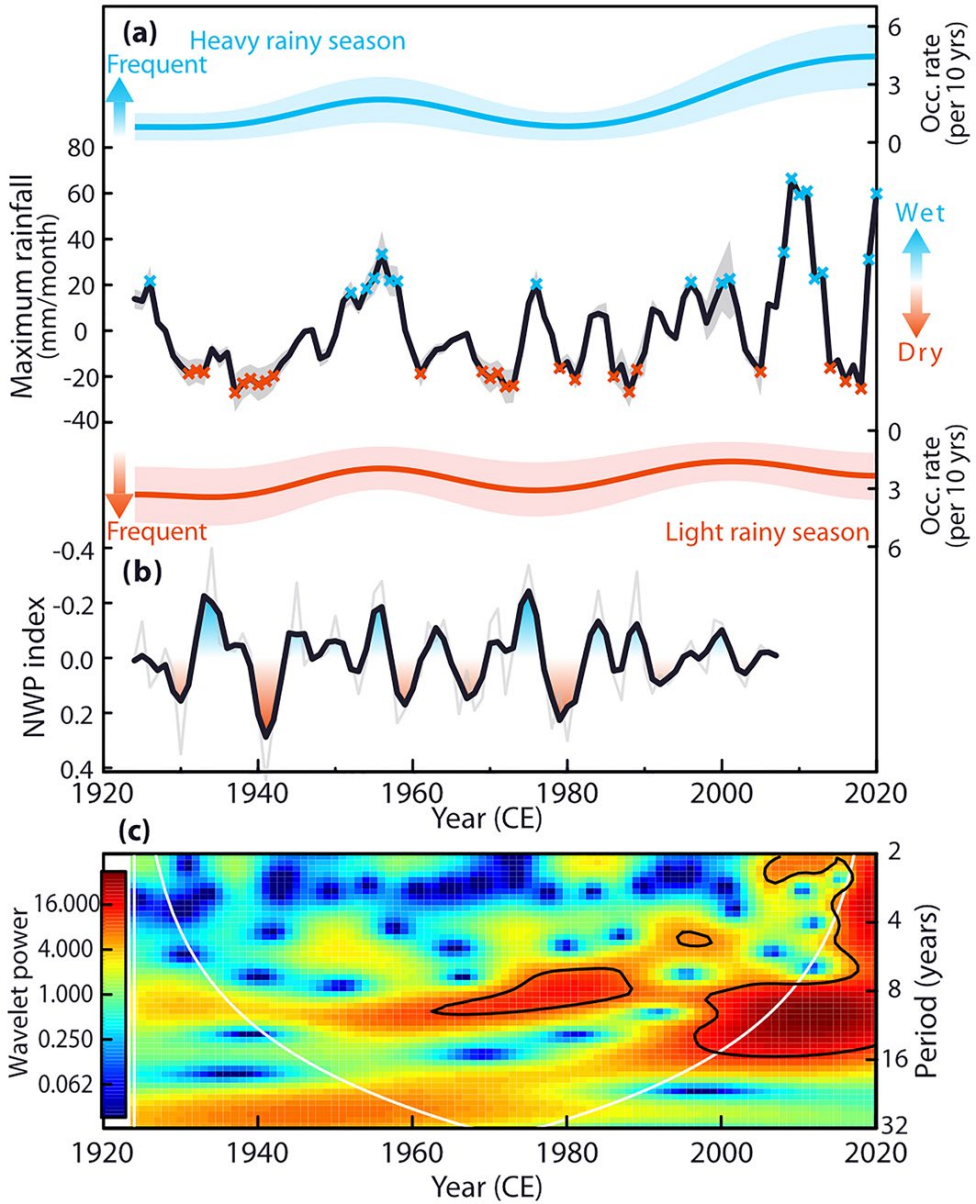
Time series analysis of the maximum rainfall. (**a**) The maximum rainfall_recon+GPCP_ (black line) derived from the min-δ^18^O_sw_ and rainfall_GPCP_. Grey shades indicate the 90% confidence intervals of maximum rainfall reconstruction. Cross plots indicate the year with the heavy (blue) or light (red) rainy season. Note that rainfall in the heavy rainy season (blue cross plot) shows an increasing trend. The occurrence rates of heavy (blue line) and light (red line) rainy seasons are indicated above and below panel a, respectively. Light-coloured shades indicate the 90% confidence bands of occurrence rates. (**b**) The NWP index^18^ (thin and thick lines: annual and 3-year moving averaged data). (**c**) A Morlet wavelet analysis for the maximum rainfall_recon+GPCP_. The solid black lines indicate a 95% confidence level. White lines show the cone of influence, where edge effects reduce the variance.

The δ^18^O_sw_ was calibrated with the combined rainfall data from observations and satellite data (derived from the Global Precipitation Climatology Project dataset, GPCP version 2.3^31^) over the MRD from 1979 to 2020 CE (Fig. 1a). The min-δ^18^O_sw_ was compared with the annual maxima of rainfall derived from the GPCP dataset (maximum rainfall_GPCP_, reported as an anomaly relative to the reference period). The annual maximum rainfall (maximum rainfall_recon_) was reconstructed following the calibration work to the min-δ^18^O_sw_ and composited (maximum rainfall_recon+GPCP_) with the maximum rainfall_GPCP_ in the MRD. Based on the maximum rainfall_recon+GPCP_ (Fig. 2a), the occurrence rates of years with rainfall beyond one standard deviation in the reference period (heavy and light rainy seasons) were estimated using the kernel technique^32^. The maximum rainfall_recon_ was compared with the coral-based indexes of the Niño warm pool (NWP) and Niño cold tongue (NCT) as indicators of ENSO in the central Pacific (CP) and eastern Pacific (EP) Oceans^18^. We investigated the teleconnection of the ENSO by comparing the maximum rainfall_recon+GPCP_ with the thermal gradient between the western (0–10°N, 130–150°E) and central Pacific Oceans (5°S–5°N, 160–210°E)^33^.

## Result and discussion

### Calibration of the coral record from Con Dao Island

The SST-related proxies of Sr/Ca and δ^18^O_coral_ showed 82 seasonal cycles from December 1923 to June 2006 (Fig. 1c). The range of calculated δ^18^O_sw_ was from –1.11 to 0.97‰. δ^18^O_sw_ during 1979–2005 CE correlated with in situ salinity on Con Dao Island and rainfall changes in the MRD over a 95% confidence level (CL) (Fig. S3; δ^18^O_sw_ vs. salinity: *R* = 0.36; *n* = 291, δ^18^O_sw_ vs. rainfall: *R* = − 0.32; *n* = 324). The min-δ^18^O_sw,_ which ranged from − 0.84 to 0.51‰, negatively correlated with the maximum rainfall_GPCP_ (annual data: *R* = − 0.37; *n* = 27, over 95% CL; 3-year moving average data: *R* = − 0.73; *n* = 27, slope: − 76.8 ± 14.2 mm × month^−1^/‰, over 99% CL) (Fig. 1d,e). The min-δ^18^O_sw_ positively correlated with SST changes in the rainy season inferred from Sr/Ca through the entire coral record (annual data: *R* = 0.52; *n* = 82; over 99% CL; 3-year moving averages: *R* = 0.50; *n* = 82; over 99% CL; Fig. S4).

Our coral-based δ^18^O_sw_ record captures year-to-year changes in maximum rainfall in the MRD (Fig. 1d,e). Our calibration agrees with previous works showing that the coral-based δ^18^O_sw_ along the southern Vietnamese coast reflects rainfall changes^34^ and significantly decreases in flood years^28^. Since the oxygen isotope ratio in raindrops (< –5‰^35,36^) is much lower than δ^18^O_sw_ (ca. 0‰^37^), river runoff and rainfall in the rainy season decrease the values of δ^18^O_sw_ in the surrounding ocean. Ocean currents (e.g., upwelling) along the southern coast also may affect salinity around Con-Dao Island. Our calibration work^28^ (Phan et al. 2017) on the Con Dao Island showed that the influence of the ocean current is smaller than rainfall in the wet season. The correlation between min-δ^18^O_sw_ and SST changes in the rainy season results from an air-sea interaction over the western Pacific Ocean^38^. Hence, the coral-based δ^18^O_sw_ records the maximum rainfall in the MRD through the entire coral time series.

### ENSO footprint in the Mekong River Delta hydrology

**Figure 3:**
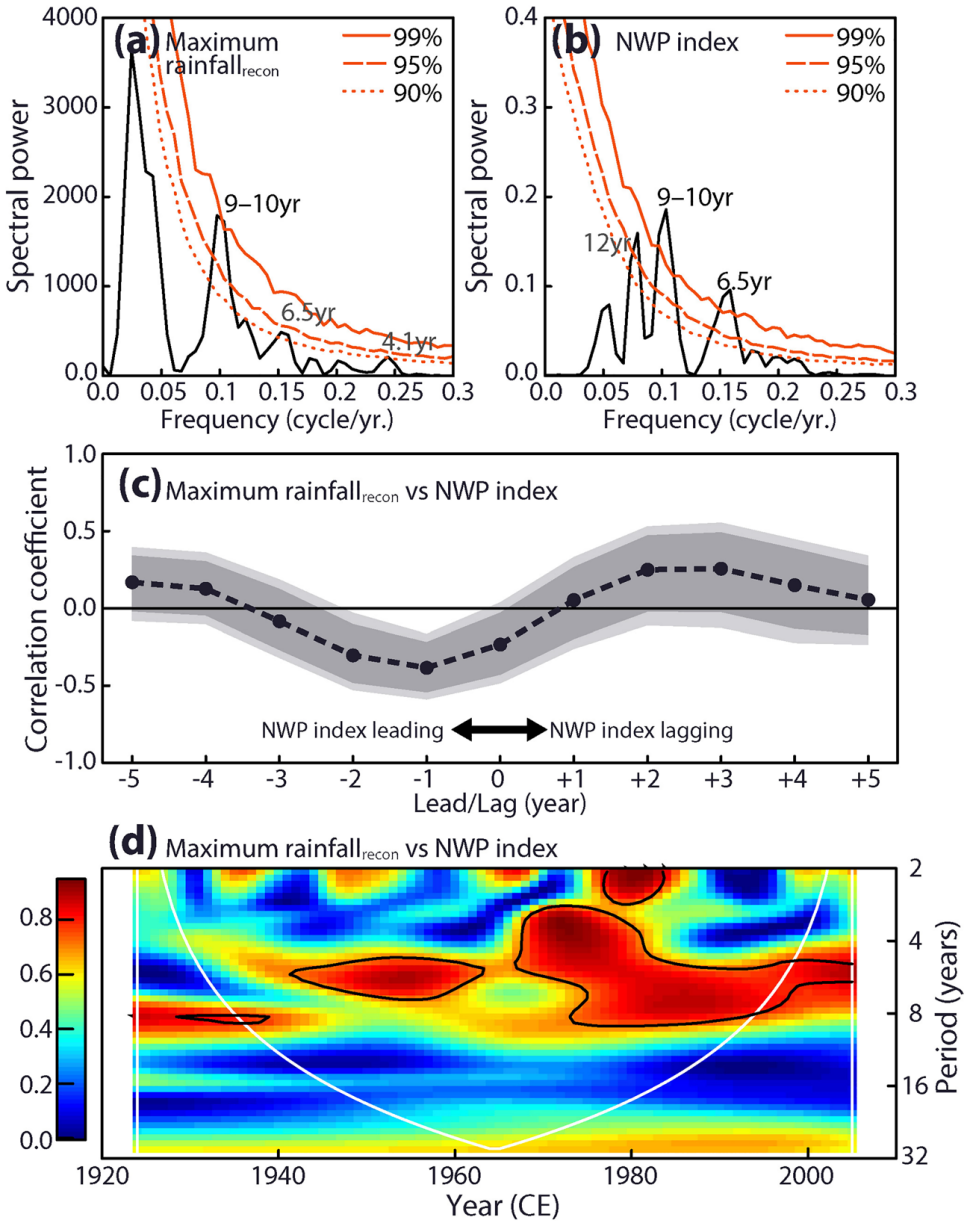
Relationship between the maximum rainfall_recon_ and the CP ENSO. (**a**,**b**) REDFIT spectral analyses^39^ for the maximum rainfall_recon_ and the NWP index. Red lines indicate bootstrap confidence intervals for the spectral power (solid: 99%; dash: 95%; dot: 90%). The REDFIT spectral analyses were performed using PAST software version 4.04^47^. (**c**) A lead-lag correlation analysis between the NWP index and the maximum rainfall_recon_. Shading areas indicate the confidence bands of correlation coefficients (light colour: 99%; thick colour: 95%). (**d**) A wavelet coherency between the maximum rainfall_recon_ and the NWP index. The solid black lines indicate a 95% confidence level. White lines show the cone of influence, where edge effects reduce the variance.

A spectral analysis^39^ to the maximum rainfall_recon_ (range: from − 27.1 to 33.4 mm/month; Fig. S5) demonstrated that the major periodicity was the interannual (4.1 and 6.5 years/cycle: over 90% CL) and decadal (9.6 years/cycle: over 99% CL; 9–10 years/cycle: over 90% CL, Fig. 3a) time scales. The major periodicity of maximum rainfall_recon_ (Fig. 3a) agreed with that of the NWP index (6.5 and 9–10 years/cycle; over 99% CL; Figs. 2b, 3b) but not the NCT index (13.6, 8.6–9, and 6.3–5 years/cycle; over 99% CL; Fig. S6). The NWP index with a one-year lead negatively correlated with the maximum rainfall_recon_ (Fig. 3c: one-year leading NWP index vs. maximum rainfall_recon_: *R* = − 0.38; *n* = 82; over 99% CL). The field correlation analysis showed the negative correlation between the detrended SST in the CP region with one year leading and the maximum rainfall_recon+GPCP_ (Fig. S7), which supported the result of the lead-lag correlation (Fig. 3c).

La Niña and CP El Niño events would have led to the heavy and light rainy seasons in the MRD, respectively (Fig. 3). Anomalous development of the low-level circulation over the western Pacific Ocean during La Niña and CP El Niño events causes anomalous rainfall in the rainy season in the MRD, as discussed in meteorology and simulations^16,40^. The development of anomalous cyclonic circulation over the western Pacific Ocean during La Niña events intensifies moisture transportation to the Indo-China Peninsula^11,17^. Cold anomalies over the Indian and central Pacific Oceans enhance the Walker circulation during La Niña events, which increases the rainfall amount in the rainy season^17^. During CP El Niño events, the rainfall amount in the rainy season decreases due to the anomalous anticyclonic circulation over the western Pacific Ocean and the weakening of the Walker circulation. CP El Niño events have a different teleconnection pattern from EP El Niño events^18,41–43^. Since the anticyclone over the western Pacific Ocean appears further west during CP El Niño events compared to EP El Niño events^37^, CP El Niño events significantly influence maximum rainfall in the MRD.

### Inconsistent trends of the hydrological events

The impacts of La Niña and CP El Niño on the frequencies and intensities of hydrological events in the MRD were not consistent throughout the past century. A wavelet analysis revealed that the major periodicity of the maximum rainfall has shifted from decadal to interannual time scales since the 1960s (Figs. 2c and S5). The periodicity shift in the maximum rainfall was observed in a wavelet coherency between the maximum rainfall_recon_ and the NWP index (Fig. 3d). These wavelet analyses (Figs. 2c, 3d, and S5) suggested that the frequent ENSO events in the CP region^18,41^ likely increased the frequency of maximum rainfall changes in the MRD in recent decades. The recent highly frequent change in the maximum rainfall_recon_ was confirmed by the increasing trends of occurrence rate and rainfall amount of the heavy rainy season throughout the last 100 years (rainfall amount: two-sided *p* < 0.01; *n* = 18; occurrence rate: one-sided *p* = 0.01; *n* = 18; Fig. 2a). In contrast to the heavy rainy season, these trends of occurrence rate and rainfall amount were not confirmed in the light rainy season (rainfall amount: two-sided *p* = 0.94; *n* = 20; occurrence rate: one-sided *p* = 0.22; *n* = 20; Fig. 2a).

**Figure 4:**
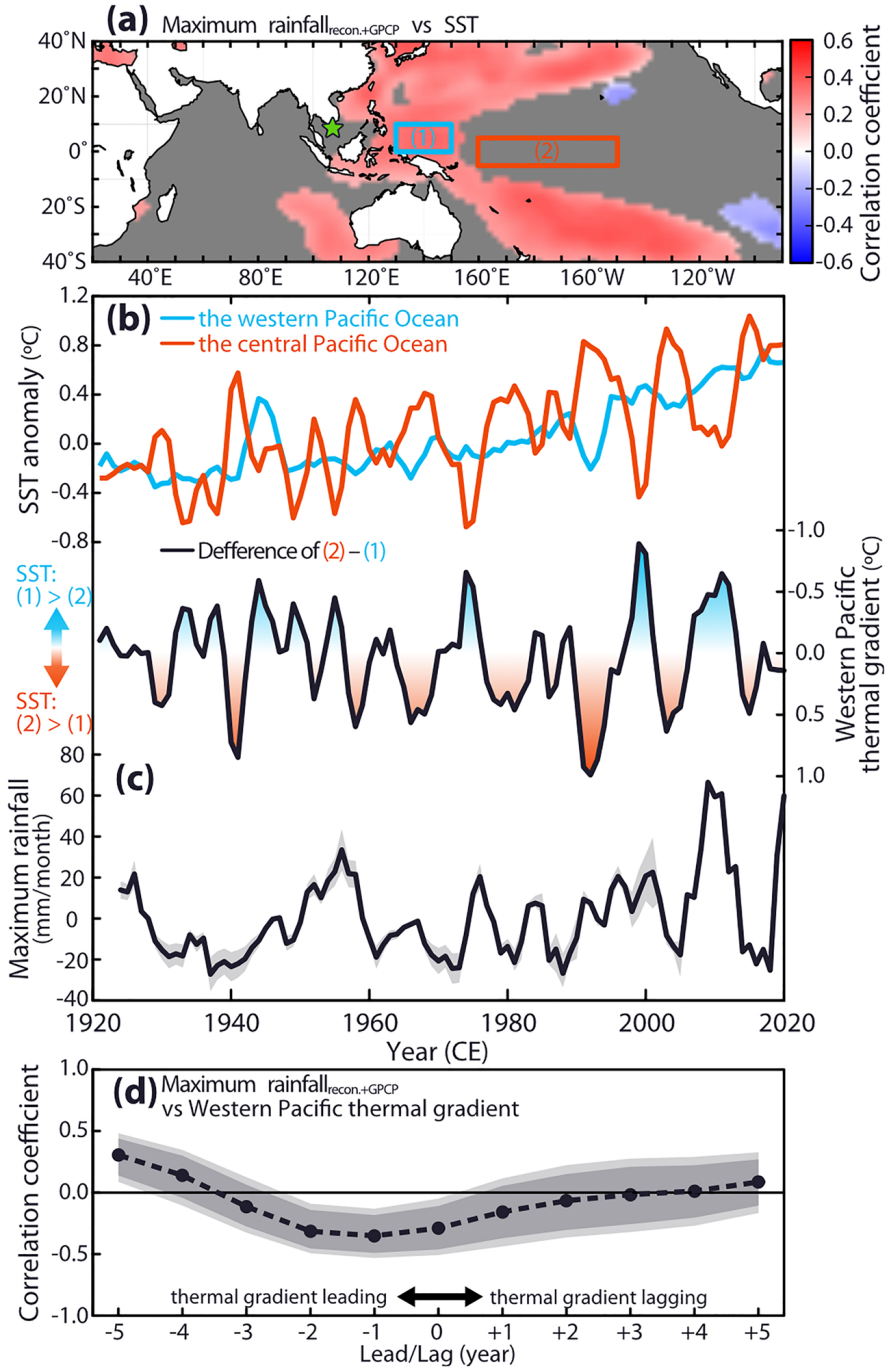
Comparing the maximum rainfall with SST in the Pacific Ocean. (**a**) A field correlation of the maximum rainfall_recon+GPCP_ and SST (Extended Reconstructed Sea Surface Temperature, ERSSTv5^48^) during 1924–2020 CE. Note the positive correlation in the western Pacific Ocean (blue box) and insignificant correlation in the CP region (red box). A green star indicates our sample site. The map was generated by the GMT 4.5.12^46^. Correlations were computed with the web application KNMI Climate explorer. Correlations not significant at the 10% level are masked out. (**b**) The upper panel indicates 3-year moving averages of the SST anomaly in the western (blue line; **a** blue box in panel **a**) and central Pacific Ocean (red line; a red box in panel **a**). SST data are derived from ERSSTv5^48^. The lower panel indicates the western Pacific thermal gradient. (**c**) The maximum rainfall_recon+GPCP_ (black line). Grey shades indicate the 90% confidence interval. (**d**) A lead-lag correlation analysis between the western Pacific thermal gradient and the maximum rainfall_recon+GPCP_. Shaded areas indicate the confidence bands of correlation coefficients (light colour: 99%; thick colour: 95%).

The correlation analyses implied that the inconsistent trends between the heavy and light rainy seasons were closely related to increases of SST in the western Pacific Ocean (*R* = 0.36; *n* = 97; over 99% CL; Figs. 4a and S8) and the western Pacific thermal gradient (*R* = − 0.29; *n* = 97; over 99% CL, Fig. 4). The warming rate in the western Pacific Ocean was faster than that in the CP region and thus has intensified the thermal gradient over the western Pacific Ocean during La Niña events since the 1980s^33^. The intensified thermal gradient during La Niña events further strengthens the Pacific Walker circulation, which amplifies heavy rainfall in the MRD during La Niña events.

## Conclusion

In reference to our coral-based δ^18^O_sw_ record, we revealed inconsistent trends of frequency and intensity between the heavy and light rainy seasons, which likely impact socioeconomics and human health in the MRD. The heavy rainy season recurs more frequently and has more serious impacts in recent decades, resulting from the more frequent occurrence of CP El Niño events and the intensified western Pacific thermal gradient. Hence, people dwelling in the MRD will face a higher risk of severe flood events. However, there was no clear trend in the occurrence rate and rainfall amount of the light rainy season, although catastrophic drought hit the MRD in 2015 and 2019 CE. We concluded that the hydrological changes in the MRD are sensitive to the CP ENSO events. The hydrological response to the ENSO behaviour is inconsistent throughout the last century, which will improve the prediction of the future impact of the flood and drought.

## Methods

### Sample preparation and proxy measurement

The coral core (CD04) was collected from Con Dao Island, located 90 km far from the MRD. The core was sliced into 5 mm thickness. X-ray images of slices were taken using a digital X-ray scanner to visualise the coral growth transaction and annual bands.

Monthly coral skeletal powdered subsamples for geochemical analyses (for Sr/Ca and δ^18^O_coral_) were drilled at ca. 1 mm intervals along the maximum axis of coral growth. δ^18^O_coral_ of powdered subsample, 20–40 μg each, was analysed using a stable isotope ratio mass spectrometer (Finnigan MAT253) and an automated carbonate device (Kiel IV) at Hokkaido University. The coral skeletal powdered subsamples, 90–110 μg, were dissolved in 25% nitric acid and diluted to a Ca concentration of 7 ppm with ultrapure water for Sr/Ca determination^48^ on an inductively coupled plasma optical emission spectrometer, Thermo Scientific iCAP 6200 at Hokkaido University. The one-sigma precisions for δ^18^O_coral_ and Sr/Ca data were ± 0.05‰_VPDB_ and ± 0.07% RSD, respectively.

We calculated the δ^18^O_sw_ value (relative to Vienna Standard Mean Ocean Water, VSMOW) based on paired measurements of Sr/Ca and δ^18^O_coral_ using the centring method^30^. Centred values of Sr/Ca and δ^18^O_coral_ (values relative to the mean of the entire records) were used for the δ^18^O_sw_ estimation to avoid the large uncertainty source for this estimation^26^. We used the slope of our Sr/Ca-SST dependency on Con Dao Island (− 0.0504 ± 0.004 mmol × mol^−1^/°C)^28^ and the slope value of the δ^18^O_coral_-SST relationship (− 0.18‰_VPDB_/ºC^30,50^). The one-sigma uncertainty of calculated δ^18^O_sw_ was ± 0.06‰. The age model for the coral records was developed using the maxima and minima of the Sr/Ca values in any annual cycle as anchoring points. The anchoring points were tied to the maxima and minima of SST on Con Dao Island^28^. We used linear interpolation to obtain monthly resolution time series using AnalySeries software^51^, version 2.0.8.

### Statistical analyses

We estimated the mean SST in the rainy season and obtained the annual minima of δ^18^O_sw_ (min-δ^18^O_sw_). Min-δ^18^O_sw_ was reported as the anomaly relative to the reference period (1981–2000 CE). The reference period was defined as the 20-year period in which the min-δ^18^O_sw_ and rainfall_GPCP_ overlapped. Our coral record was compared with rainfall_GPCP_ (7.5–15°N, 105–110°E, green box in Fig. 1a), the NWP index (index for the CP ENSO)^18^, and the NCT index (index for the EP ENSO)^18^. We estimated the annual rainfall maxima (maximum rainfall_recon,_ values relative to 1981–2000 CE) from the 3-year moving average of the min-δ^18^O_sw_ based on the slope between the 3-year moving average datasets of min-δ^18^O_sw_ and the maximum rainfall_GPCP_ (− 76.8 ± 14.2 mm × month^−1^/‰, Fig. 1e). The uncertainty of maximum rainfall_recon+GPCP_ was estimated using the Monte Carlo approach. We considered an error for the slope between the min-δ^18^O_sw_ versus maximum rainfall_GPCP_ and the differences between maximum rainfall_GPCP_ versus maximum rainfall_recon_. We repeatedly estimated maximum rainfall by adding random numbers known from the slope errors in the 20,000 times loop.

We estimated the periodicity using REDFIT spectral analysis^39^ in PAST software version 4.04^47^. The correlation coefficient and confidence level were estimated using the bootstrap technique and the BCa method in the “wBoot” package in R software^52^. The occurrence rates of the heavy/light rainy season were estimated using the kernel technique in Caliza software^32^. The heavy/light rainy season was defined as a year with maximum rainfall beyond the threshold (± 15.4 mm/month). The threshold value was estimated as the average value plus/minus one standard deviation in maximum rainfall_recon+GPCP_ during the reference period (1981–2000 CE). The bandwidth for the estimation of the occurrence rate was set to 11 years to detect decadal-scale variations. The confidence interval of the occurrence rate was determined using a bootstrap technique and a percentile-t method. Events were simulated with replacement and used for occurrence rate calculations, which were repeated 5000 times. Wavelet analyses were conducted using the “biwavelet” package in R software^52^. We surveyed the significance of trends in the occurrence rate and rainfall amount of the heavy/light rainy season based on the Cox-Lewis test in Caliza software^32^ and the Mann–Kendall trend test in R software^52^, respectively.

## Supplementary Information

Supplementary Information.

## References

[1] Clauss K, Ottinger M, Leinenkugel P, Kuenzer C (2019). Estimating rice production in the Mekong Delta, Vietnam, utilizing time series of Sentinel-1 SAR data. Int. J. Appl. Earth Obs. Geoinf..

[2] Tan Yen, B. et al. Modeling ENSO impact on rice production in the Mekong River Delta. *PloS ONE* **14**(10), e0223884. 10.1371/journal.pone.0223884 (2019). 10.1371/journal.pone.0223884 PMC6804992 31639159

[3] Kang H, Sridhar V, Mainuddin M (2021). Future rice farming threatened by drought in the Lower Mekong Basin. Sci. Rep..

[4] Minderhoud PSJ, Coumou L, Erkens G, Middelkoop H, Stouthamer E (2019). Mekong delta much lower than previously assumed in sea-level rise impact assessments. Nat. Commun..

[5] Nguyen, N. A. Historic drought and salinity intrusion in the Mekong Delta in 2016: Lessons learned and response solutions. *Vietnam J. Sci. Technol. Eng.* **59**(1), 93–96. 10.31276/VJSTE.59(1).93 (2017).

[6] International Federation of Red Cross And Red Crescent Societies. Vietnam—Drought and Saltwater Intrusion Emergency Plan of Action (EPoA) DREF operation n° MDRVN019 Final Report. 1–18 (2020).

[7] Sabo, J. L. et al. Designing river flows to improve food security futures in the Lower Mekong Basin. *Science* **358**, (2017). 10.1126/science.aao1053 29217541

[8] Pokhrel Y (2018). A review of the integrated effects of changing climate, land use, and dams on Mekong river hydrology. Water.

[9] Phung D (2021). Hydropower dams, river drought and health effects: A detection and attribution study in the lower Mekong Delta Region. Clim. Risk Manag..

[10] Li G, Gao C, Lu B, Chen H (2021). Inter-annual variability of spring precipitation over the Indo-China Peninsula and its asymmetric relationship with El Niño-Southern Oscillation. Clim. Dyn..

[11] Wang B (2009). Multi-scale climate variability of the South China Sea monsoon: A review. Dyn. Atmos. Oceans.

[12] Buckley, B. M. et al. Central Vietnam climate over the past five centuries from cypress tree rings. *Clim. Dyn.* **48**(11), 3707–3723. 10.1007/s00382-016-3297-y (2017)

[13] Delgado JM, Merz B, Apel H (2012). A climate-flood link for the lower Mekong River. Hydrol. Earth Syst. Sci..

[14] Hetch J, Lacombe G, Arias ME, Dang TD, Piman T (2019). Hydropower dams of the Mekong River basin: A review of their hydrological impacts. J. Hydrol..

[15] Räsänen TA, Lindgren V, Guillaume JH, Buckley BM, Kummu M (2016). On the spatial and temporal variability of ENSO precipitation and drought teleconnection in mainland Southeast Asia. Clim. Past.

[16] Dang, V. H. et al. Assessment of rainfall distributions and characteristics in coastal provinces of the Vietnamese Mekong Delta under climate change and ENSO processes. *Water* **12**(6). https://www.mdpi.com/2073-4441/12/6/1555 (2020).

[17] Ge, F. et al. Precipitation over Indochina during the monsoon transition: modulation by Indian Ocean and ENSO regimes. *Clim. Dyn. *1–14. 10.1007/s00382-021-05817-6 (2021).

[18] Freund MB (2019). Higher frequency of Central Pacific El Niño events in recent decades relative to past centuries. Nat. Geosci..

[19] Xu C, Sano M, Nakatsuka T (2013). A 400-year record of hydroclimate variability and local ENSO history in northern Southeast Asia inferred from tree-ring δ^18^O. Palaeogeogr. Palaeoclimatol. Palaeoecol..

[20] Cook ER (2010). Asian monsoon failure and megadrought during the Last Millennium. Science.

[21] Morimoto M (2002). Salinity records for the 1997–98 El Niño from Western Pacific corals. Geophys. Res. Lett..

[22] Shen C-C (2005). An evaluation of quantitative reconstruction of past precipitation records using coral skeletal Sr/Ca and δ18O data. Earth Planet. Sci. Lett..

[23] Cahyarini SY (2014). Twentieth century sea surface temperature and salinity variations at Timor inferred from paired coral δ18O and Sr/Ca measurements. J. Geophys. Res. Oceans.

[24] Ito S, Watanabe T, Yano M, Watanabe TK (2020). Influence of local industrial changes on reef coral calcification. Sci. Rep..

[25] Krawczyk H (2020). Corals reveal ENSO-driven synchrony of climate impacts on both terrestrial and marine ecosystems in northern Borneo. Sci. Rep..

[26] Nurhati IS, Cobb KM, Di Lorenzo E (2011). Decadal-scale SST and salinity variations in the central tropical Pacific: Signatures of natural and anthropogenic climate change. J. Clim..

[27] Tran, D.D., et al. Long-term sustainability of the Vietnamese Mekong Delta in question: An economic assessment of water management alternatives. *Agric. Water Manag.* **223**. 10.1016/j.agwat.2019.105703 (2019).

[28] Phan TT (2019). Mekong River discharge and the East Asian monsoon recorded by a coral geochemical record from Con Dao Island Vietnam. Geochem. J..

[29] Shen C-C (1996). The calibration of *D*[Sr/Ca]versus sea surface temperature relationship for *Porites* corals. Geochim. Cosmochim. Acta.

[30] Cahyarini SY, Pfeiffer M, Timm O, Dullo W-C, Schönberg DG (2008). Reconstructing seawater δ18O from paired coral δ18O and Sr/Ca ratios: Methods, error analysis and problems, with examples from Tahiti (French Polynesia) and Timor (Indonesia). Geochim. Cosmochim. Acta.

[31] Adler, R. F. et al. The Global Precipitation Climatology Project (GPCP) monthly analysis (new version 2.3) and a review of 2017 global precipitation. *Atmosphere* **9**(4), 138. 10.3390/atmos9040138 (2018). 10.3390/atmos9040138 PMC6043897 30013797

[32] Mudelsee M, Börngen M, Tetzlaff G, Grünewald U (2003). No upward trends in the occurrence of extreme floods in central Europe. Nature.

[33] Hoell A, Funk C (2013). The ENSO-related west Pacific sea surface temperature gradient. J. Clim..

[34] Bolton A (2014). Paired *Porites* coral Sr/Ca and δ18O from the western South China Sea: Proxy calibration of sea surface temperature and precipitation. Palaeogeogr. Palaeoclimatol. Palaeoecol..

[35] Duy NL, Heidbüchel I, Meyer H, Merz B, Apel H (2018). What controls the stable isotope composition of precipitation in the Mekong Delta? A model-based statistical approach. Hydrol. Earth Syst. Sci..

[36] Wolf A, Roberts WH, Ersek V, Johnson KR, Griffiths ML (2020). Rainwater isotopes in central Vietnam controlled by two oceanic moisture sources and rainout effects. Sci. Rep..

[37] Schmidt GA (1999). Forward modeling of carbonate proxy data from planktonic foraminifera using oxygen isotope tracers in a global ocean model. Paleoceanography.

[38] Chen J, Wang X, Zhou W, Wen Z (2018). Interdecadal change in the summer SST-precipitation relationship around the late 1990s over the South China Sea. Clim. Dyn..

[39] Schulz M, Mudelsee M (2002). REDFIT: estimating red-noise spectra directly from unevenly spaced paleoclimatic time series. Comput. Geosci..

[40] Yuan Y, Yang S, Zhang Z (2012). Different evolutions of the Philippine Sea anticyclone between the eastern and central Pacific El Niño: Possible effects of Indian Ocean SST. J. Clim..

[41] Ashok K, Behera SK, Rao SA, Weng H, Yamagata TE (2007). Niño Modoki and its possible teleconnection. J. Geophys. Res. Oceans.

[42] Karori MA, Li J, Jin F-F (2013). The asymmetric influence of the two types of El Niño and La Niña on summer rainfall over southeast China. J. Clim..

[43] Liu Y (2017). Recent enhancement of central Pacific El Niño variability relative to last eight centuries. Nat. Commun..

[44] Kug J-S, Jin F-F, An S-I (2009). Two types of El Niño events: Cold tongue El Niño and warm pool El Niño. J. Clim..

[45] Good SA, Martin MJ, Rayner NA (2013). EN4: Quality controlled ocean temperature and salinity profiles and monthly objective analyses with uncertainty estimates. J. Geophys. Res. Oceans.

[46] Wessel P, Smith WHF (1998). New, improved version of generic mapping tools released. EOS Trans. Am. Geophys. Union.

[47] Hammer Ø, Harper DA, Ryan PD (2001). PAST: Paleontological statistics software package for education and data analysis. Palaeontol. Electron..

[48] Huang B (2017). Extended reconstructed sea surface temperature, version 5 (ERSSTv5): Upgrades, validations, and intercomparisons. J. Clim..

[49] Watanabe TK, Watanabe T, Ohmori K, Yamazaki A (2020). Improving analytical method of Sr/Ca ratios in coral skeletons for paleo-SST reconstructions using ICP-OES. Limnol. Oceanogr. Methods.

[50] Gagan MK (1998). Temperature and surface-ocean water balance of the mid-Holocene tropical western Pacific. Science.

[51] Paillard D, Labeyrie L, Yiou P (1996). Macintosh Program performs time-series analysis. EOS Trans. Am. Geophys. Union.

[52] R Core Team. R: A language and environment for statistical computing. R Foundation for Statistical Computing. Vienna, Austria, https://www.R-project.org/ (2020).

