# Supplementary material for: Nonstationary footprints of ENSO in the Mekong River Delta hydrology
Source: Sci Rep. 2022 Dec 7;12:21186. doi: 10.1038/s41598-022-20597-7 (PMC9729578; doi:10.1038/s41598-022-20597-7)
Supplement: Supplementary file 1 — Supplementary Information. [file 41598_2022_20597_MOESM1_ESM.docx]

**Supplementary information**

**
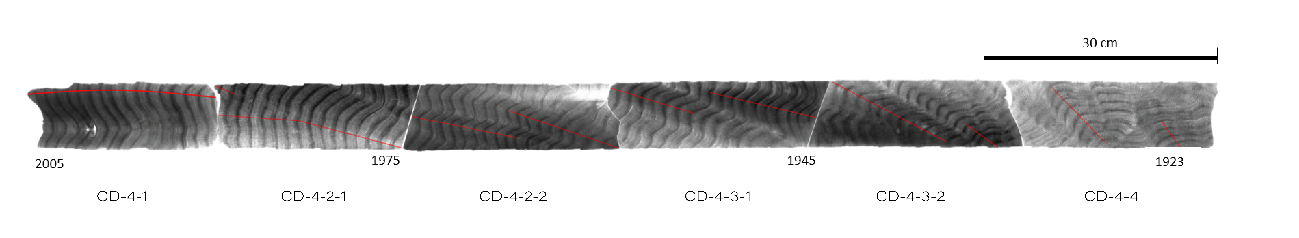
**

**Figure S1 X-ray image of the coral core (CD-4) from Con-Dao Island.**

The black bar shows a scale (30cm). The red lines indicate the maximum coral growth axis, selected as a measurement line.

**
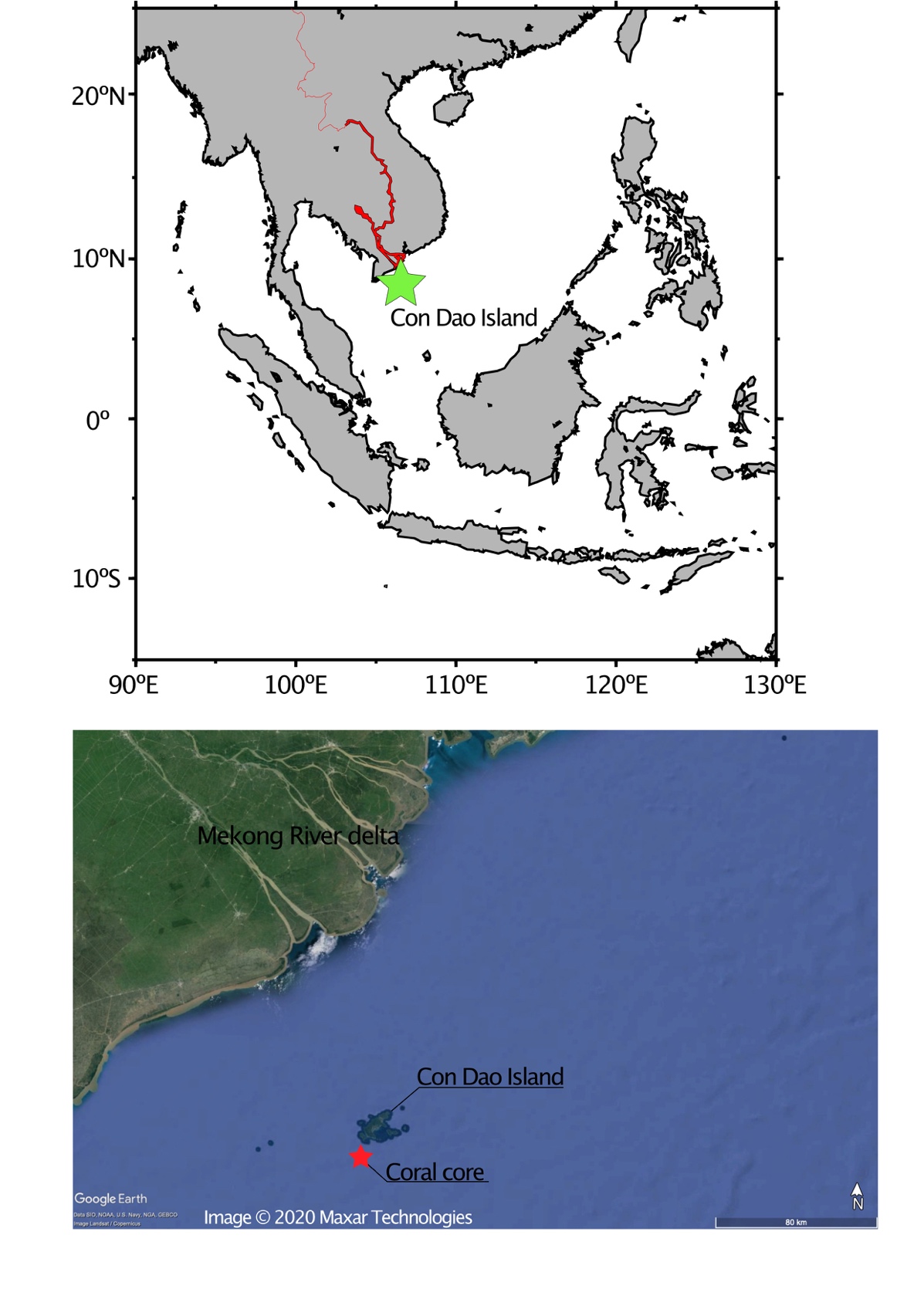
**

**Figure S2 Location of the coral core from Con-Dao Island.**

The upper panel shows the map of the south-eastern Asia. The map was generated by the GMT 4.5.12. The lower panel shows the satellite view around Con-Dao island. The star symbol indicates the location of our coral core. The satellite image is obtained from Google Earth, ©2020 Maxar Technologies. The map was modified using Adobe Illustrator 2021 version 25.0.1.

**
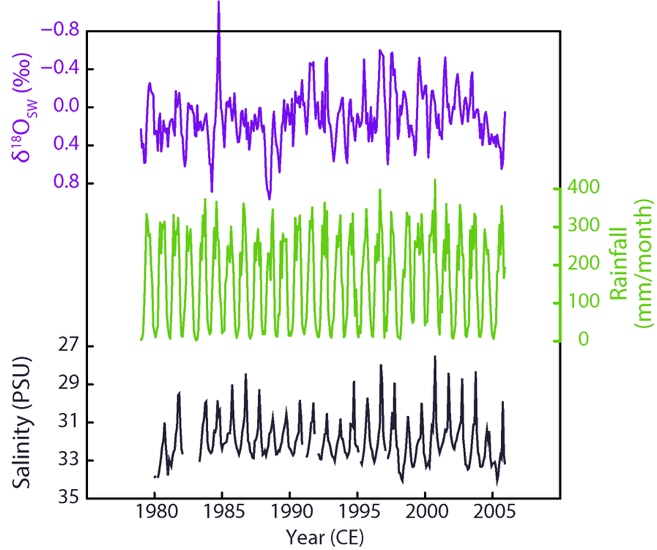
**

**Figure S3 Coral-based records and in situ data.**

Monthly-resolution coral records of δ^18^O_SW_ (purple) from 1979 to 2005 in Con Dao Island. GPCP rainfall data in the Mekong River delta (green; 7.5 – 15ºN, 100 – 110ºE, green box in Figure 1-a). *In situ* salinity at Con Dao Island^1^ (black; Phan et al., 2019).

**
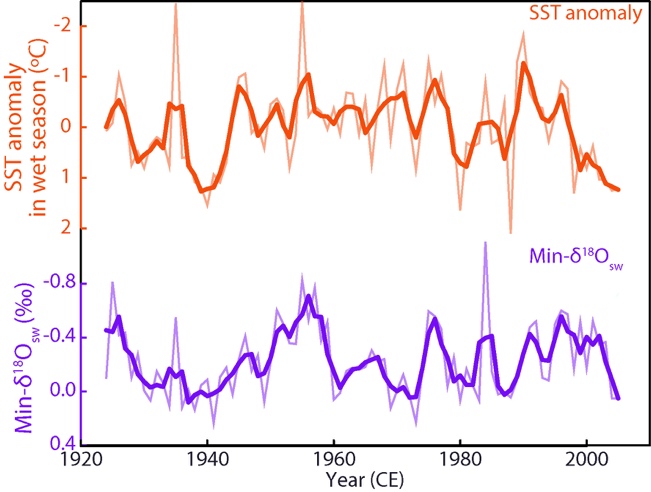
**

**Figure S4 Comparison of the coral records.**

(a) The red and black lines indicate the Sr/Ca and ERSST data around the ConDao island. (b) The red and purple lines indicate the SST anomaly in the wet season and the min-δ^18^O_sw_, respectively (thin line: original data; thick line: 3-years moving average).

**
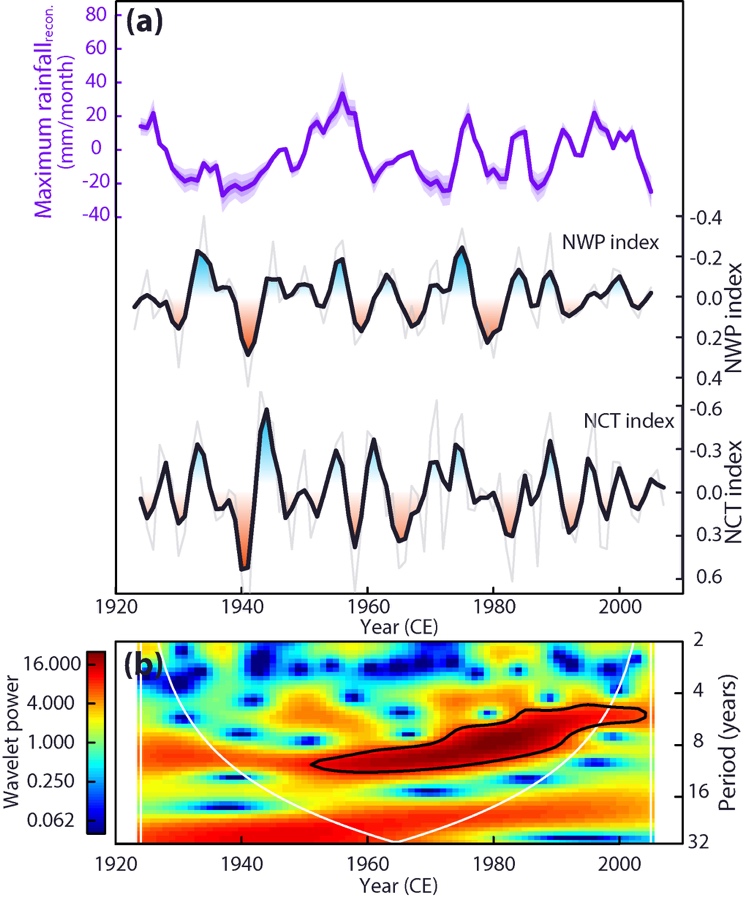
**

**Figure S5 Comparison of the maximum rainfall_recon_ with the NWP and NCT indexes.**

(a) The purple line indicates the maximum rainfall_recon_. Shades indicate 90% confidence intervals of maximum rainfall reconstruction. The black line indicates the 3-years moving average of the NWP and NCT indexes^2^ (grey line: annual data). (b) A Morlet wavelet analysis for the maximum rainfall_recon_. The solid black lines indicate a 95% confidence level. White lines show the cone of influence, where edge effects reduce the variance.


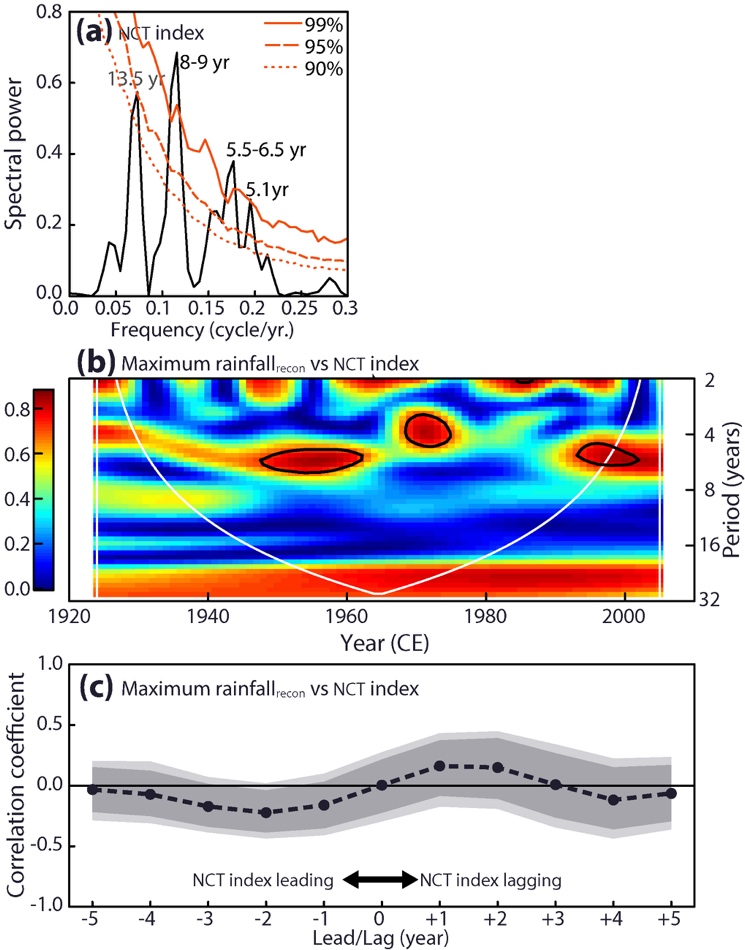


**Figure S6** **Relationship between the maximum rainfall and the NCT index.**

(a) A REDFIT spectral analysis^3^ for the NCT index. Red lines indicate bootstrap confidence interval for the spectral power (solid: 99%; dash: 95%; dot: 90%). The spectral analysis was performed using PAST software^4^ version 4.04. (b) A wavelet coherency between the maximum rainfall_recon_ and the NCT index. (c) A lead and lag correlation analysis between the NCT index and the maximum rainfall_recon._ Shading areas indicate the confidence limit of correlation coefficients (light colour: 99%; thick colour: 95%).


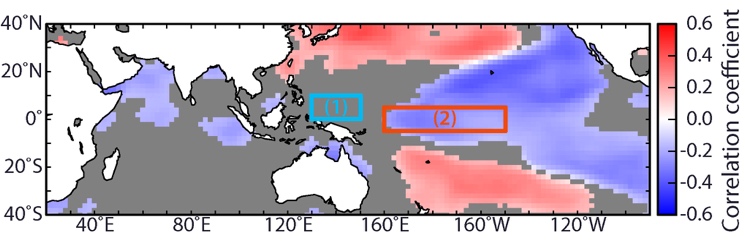


**Figure S7 Field correlation of the maximum rainfall and the detrended SST**

A field correlation of the detrended SST (ERSSTv5^5^) from 1924–2020 with a one-year lead and the maximum rainfall_recon+GPCP_. Note, the negative correlation in the central Pacific Ocean (red box). Correlations were computed with the web application KNMI Climate explorer and plotted using the GMT software^6^. Correlations less significant at the 10 % level are masked out.


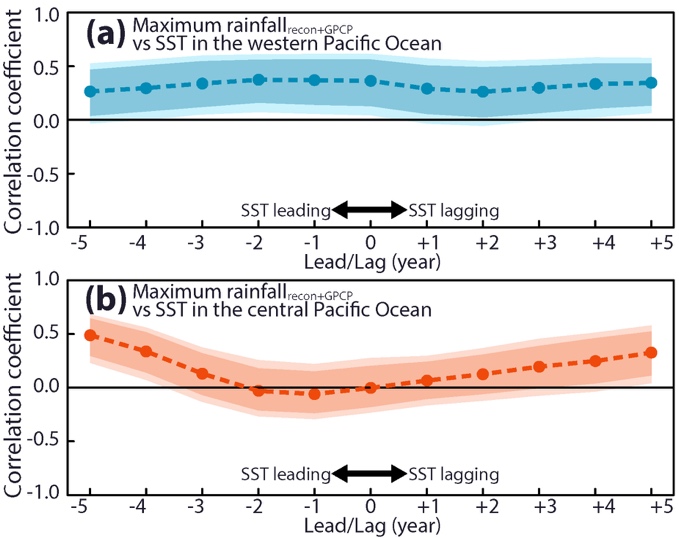


**Figure S8 Lead-lag correlations between the maximum rainfall and the Pacific SST**

Lead and lag correlations between the maximum rainfall_recon+GPCP_ and SST in the western (a) and central (b) Pacific Ocean. Shading areas indicate the confidence limit of correlation coefficients (light colour: 99%; thick colour: 95%).

**Reference list**

1. Phan, T. T. et al. Mekong River discharge and the East Asian monsoon recorded by a coral geochemical record from Con Dao Island, Vietnam. *Geochem. J.* **53**(2), e1-e7, <https://doi.org/10.2343/geochemj.2.0552> (2019).
2. Freund, M. B. et al. Higher frequency of Central Pacific El Niño events in recent decades relative to past centuries. *Nature Geoscience* **12**(6), 450-455, <https://doi.org/10.1038/s41561-019-0353-3> (2019).
3. Schulz, M. & Mudelsee, M. REDFIT: estimating red-noise spectra directly from unevenly spaced paleoclimatic time series. *Computers & Geosciences* **28**(3), 421-426, <https://doi.org/10.1016/S0098-3004(01)00044-9> (2002).
4. Hammer, Ø., Harper, D. A. & Ryan, P. D. PAST: Paleontological statistics software package for education and data analysis. *Palaeontologia Electronica* **4**(1), 9 (2001).
5. Huang, B. et al. Extended reconstructed sea surface temperature, version 5 (ERSSTv5): upgrades, validations, and intercomparisons. *Journal of Climate* **30**(20), 8179-8205, <https://doi.org/10.1175/JCLI-D-16-0836.1> (2017).
6. Wessel, Paul, and Walter HF Smith. New, improved version of Generic Mapping Tools released. *Eos, Transactions American Geophysical Union* **79**(47) 579-579, https://doi.org/10.1029/98EO00426 (1998)
